# Correlation between the production of exopolysaccharides and oxalic acid secretion by *Ganoderma applanatum* and *Tyromyces palustris*

**DOI:** 10.1007/s11274-014-1733-x

**Published:** 2014-09-02

**Authors:** Monika Osińska-Jaroszuk, Kamila Wlizło, Katarzyna Szałapata, Anna Jarosz-Wilkołazka

**Affiliations:** Department of Biochemistry, Maria Curie-Sklodowska University, Akademicka Street 19, 20-033 Lublin, Poland

**Keywords:** Exopolysaccharides, Oxalic acid, Tyromyces palustris, Ganoderma applanatum

## Abstract

The secretion of exopolysaccharides and oxalic acid in cultures of a white rot *Ganoderma applanatum* strain and a brown rot *Tyromyces palustris* strain were tested in terms of culture time, pH range, and temperature. The high yield of exopolysaccharides (EPS) required a moderate temperature of 28 °C for *G. applanatum* and 20 °C for *T. palustris.* *G. applanatum* and *T. palustris* accumulated more EPS when the concentration of the carbon source (maltose for *G. applanatum* and fructose for *T. palustris*) was 30 g/L. The results indicate that the production of oxalic acid by *G. applanatum* is correlated with the initial pH value of the culture medium and the concentration of oxalic acid increased to 1.66 ± 0.2 mM at the initial pH of 6.5 during the fungal growth. During the growth of *T. palustris*, the reduction of the initial pH value of the growing medium lowered the oxalic acid concentration from 7.7 ± 0.6 mM at pH 6.0 to 1.99 ± 0.2 mM at pH 3.5. *T. palustris* accumulated considerably more oxalic acid than *G. applanatum* and its presence did not affect significantly the production of exopolysaccharides. We also observed that the maximum amounts of exopolysaccharides secreted during cultivation of *G. applanatum* and *T. palustris* were 45.8 ± 1.2 and 19.1 ± 1.2 g/L, respectively.

## Introduction

For thousands of years, mushrooms have been valued as edible and medicinal resources. A number of polysaccharides isolated from fungi, yeasts, and other microbial strains have been studied and used for pharmaceutical purposes such as dietary supplements and drugs (Zhang et al. 2007; Chen and Seviour 2007; Danot et al. 2012). Fruiting bodies are the main source of biological active fungal compounds; however, it usually takes several months to obtain them. Because of this, fungal submerged cultivations carried out to obtain extracellular active compounds have received great interest. This type of cultures may be affected by many parameters such as the initial medium pH, growing temperature, medium composition which can regulate fungal morphology and structure, the uptake of various nutrients, and the biosynthesis of the active product.

*Ganoderma applanatum,* a basidiomycetous fungus belonging to the family Polyporaceae, is used in traditional Chinese medicine as an anti-cancer agent (Usui et al. 1983). Polysaccharides extracted from fruiting bodies and mycelium of *G. applanatum* have antitumor, antiviral, and immunostimulating activities (Usui et al. 1983; Lee et al. 2007). In its natural environment, *G. applanatum* is known as a white rot fungus degrading both polysaccharides and lignin in wood (Martinez et al. 2005). *Tyromyces palustris* belongs to the ecological group of brown rot fungi accumulating organic acid, mainly oxalic acid, in its surroundings, causing a rapid decrease in pH values (Sakai et al. 2006). In the brown rot wood decay system, oxalic acid can take part in enzymatic and non-enzymatic hydrolysis of carbohydrates and as a metal chelator (Shimada et al. 1997). White rot fungi do not accumulate the acid to such a great extent as brown rot fungal strains, but rather decompose it to carbon dioxide and other products (Hirano et al. 1995; Mäkelä et al. 2010). Recently, some white rot fungal strains have been reported to overexcrete oxalic acid (Grąz et al. 2009; Sullivan et al. 2012). Oxalic acid production in white rot fungi is involved in depolymerisation of lignocellulose by producing radical species, in buffering of the fungal microenvironment, in regulation of oxidative enzyme activities, and in chelation of metals (Shimada et al. 1997; Jarosz-Wilkolazka and Graz 2006).

There have been numerous reports on the structural characterization and biological activities of fungal endopolysaccharides isolated from mycelial biomass and fruiting bodies (Zhang et al. 2007; Danot et al. 2012; Kozarski et al. 2011). However, there is little information about the production of extracellular polysaccharides by basidiomycetous fungi, their properties and the correlation of secretion thereof with culture parameters. Several studies have shown that exopolysaccharides were produced in liquid cultures of yeasts and ascomycetous and basidiomycetous fungal strains such as *Cordyceps sinensis, Ganoderma applanatum, Antrodia camphorata, Sclerotium sp.*, or *Schizophyllum commune* (Danot et al. 2012; Lee et al. 2007; Shu and Lung 2004; Leung et al. 2009). These exopolysaccharides very often have bioactive properties and differ in their structural complexities (Leung et al. 2009). The production of exopolysaccharides and their physico-chemical compositions depend on the culture conditions, for example pH values, medium composition, growing temperatures, or aeration conditions of cultures (Shu and Lung 2004).

Although oxalic acid formation and its possible biochemical roles in white rot and brown rot fungi has been examined (Shimada et al. 1997), little is known about its role in exopolysaccharide synthesis by these fungi. The aim of this work was to investigate the influence of oxalic acid secretion on exopolysaccharide production in two ecologically different fungal strains, i.e. *Ganoderma applanatum* (white rot strain) and *Tyromyces palustris* (brown rot strain). Recently, an exopolysaccharide isolated from *G. applanatum* has been shown to have many biomedical, e.g. anti-cancer, immunomodulating, and antibacterial properties (Osińska-Jaroszuk et al. 2014). Therefore, the knowledge about the production and secretion of this exopolysaccharide should be extended to find out fungal strains with extraordinary physiological and biotechnological properties.

## Materials and methods

### Fungal strains and culture conditions

*Ganoderma applanatum* and *Tyromyces palustris* were obtained from the Fungal Collection of the Biochemistry Department, Maria Curie-Sklodowska University, Lublin, Poland. The cultures were maintained on 2 % synthetic potato dextrose agar (PDA) plates, which were inoculated and incubated at 25 °C for 7 days, and then stored at 4 °C as seeds for shake-flask fermentation. *Inocula* were prepared in 100-mL Elenmeyer flasks containing 25 mL of 2 % synthetic potato dextrose medium at 25 °C for 7 days; next they were homogenized and used for culture inoculation (4 % v/v). 250-mL Elenmeyer flasks contained 100 mL medium consisting of the following components (g/L): glucose 30, (NH_4_)_2_SO_4_ 1, KH_2_PO_4_ 0.5, MgSO_4_ × 7 H_2_O 0.5, FeSO_4_ × 7 H_2_O 0.01, and yeast extract 1. Furthermore, to study the optimization of the carbon source, different sugars such as fructose, maltose, sucrose, and lactose were used. The medium pH was adjusted to appropriate values by adding 1 M NaOH or 1 M HCl. The experiments were performed at 28 °C on a rotary shaker incubator at 120 rpm for 14 days. During the cultivation period, the pH value, the production of exopolysaccharides, the amount of organic acids, and the concentration of residual sugars were monitored in the culture medium.

### Analytical methods

#### Evaluation of biomass and exopolysaccharide production

The fungal mycelium was separated from the fermentation broth by filtration through a Miracloth. The mycelia were washed three times with distilled water, dried to a constant mass for 4 h at 80 °C, and then dry weights of fungal biomass were measured. The fermentation broth filtrates were collected and crude exopolysaccharides (EPS) were precipitated with the addition of four volumes of 95 % ethanol. The precipitated exopolysaccharides were collected by centrifugation at 8,000 rpm for 10 min, dried at 60 °C to remove residual ethanol, and weighted.

#### Determination of the total carbohydrate content and reducing sugars

The total carbohydrate content of the polysaccharide was examined using the phenol–sulphuric acid colorimetric method with glucose as the standard (Dubois et al. 1956). The reducing sugar was determined by the Somogyi-Nelson colorimetric method based on the procedure described by Hope and Burns (1987). The amount of total polysaccharides was calculated as the difference between total carbohydrates and reducing sugars.

#### Determination of protein, hexosamine, and uronic acids

The protein content was analysed according to Bradford (1976) using bovine serum albumin as the standard. Uronic acids in the polysaccharide was quantified by the borate methods (Biter and Muir 1962) using galacturonic acid as the standard. The hexosamine content was determined by the methods of Elson–Morgan (Belcher et al. 1954) with the para-(dimethylamino) benzaldehyde (PDAB) reagent using glucosamine as the standard.

#### Determination of organic acids using capillary electrophoresis

Capillary electrophoresis was performed with a Thermo Capillary Electrophoresis Crystal 100 instrument (Thermo Separation Products, San Jose, USA) equipped with a UV–Vis diode array detector. Separations were accomplished in a fused silica capillary with the total length of 75 cm (50 cm to the detection window). The instrument was operated at −22 kV and the capillary temperature was maintained at 25 °C. Samples were introduced by hydrodynamic injection for 0.5 s and organic acid detection was performed at 210 nm. The buffer solution (pH 6.6) was prepared according to Chen et al. (1997) and contained phthalic acid, cetyltrimethylammonium bromide (CTAB), and methanol in MilliQ water. Identification of peaks was done by spiking with commercially available standards of organic acids (formic, oxalic, malic, malonic, succinic, and tartaric acids).

#### FT-IR spectroscopy analysis

To determine the composition of the exopolysaccharides, complete acid hydrolysis thereof was carried out with 4.95 N trifluoroacetic acid (TFA) at 80 °C in a heating block for 4 h. The mixture was cooled to room temperature, evaporated, and then analysed with infrared spectroscopy. FT-IR spectroscopy was recorded with a spectrometer (Thermo Scientific Nicolet 8700A with an FT Ramana Nicolet NXR module) in the wavelength range 4,000–400 cm^−1^.

## Results

### Effect of culture conditions on the production of fungal biomass, exopolysaccharides, and oxalic acid

#### Effect of culture time

**Fig. 1:**
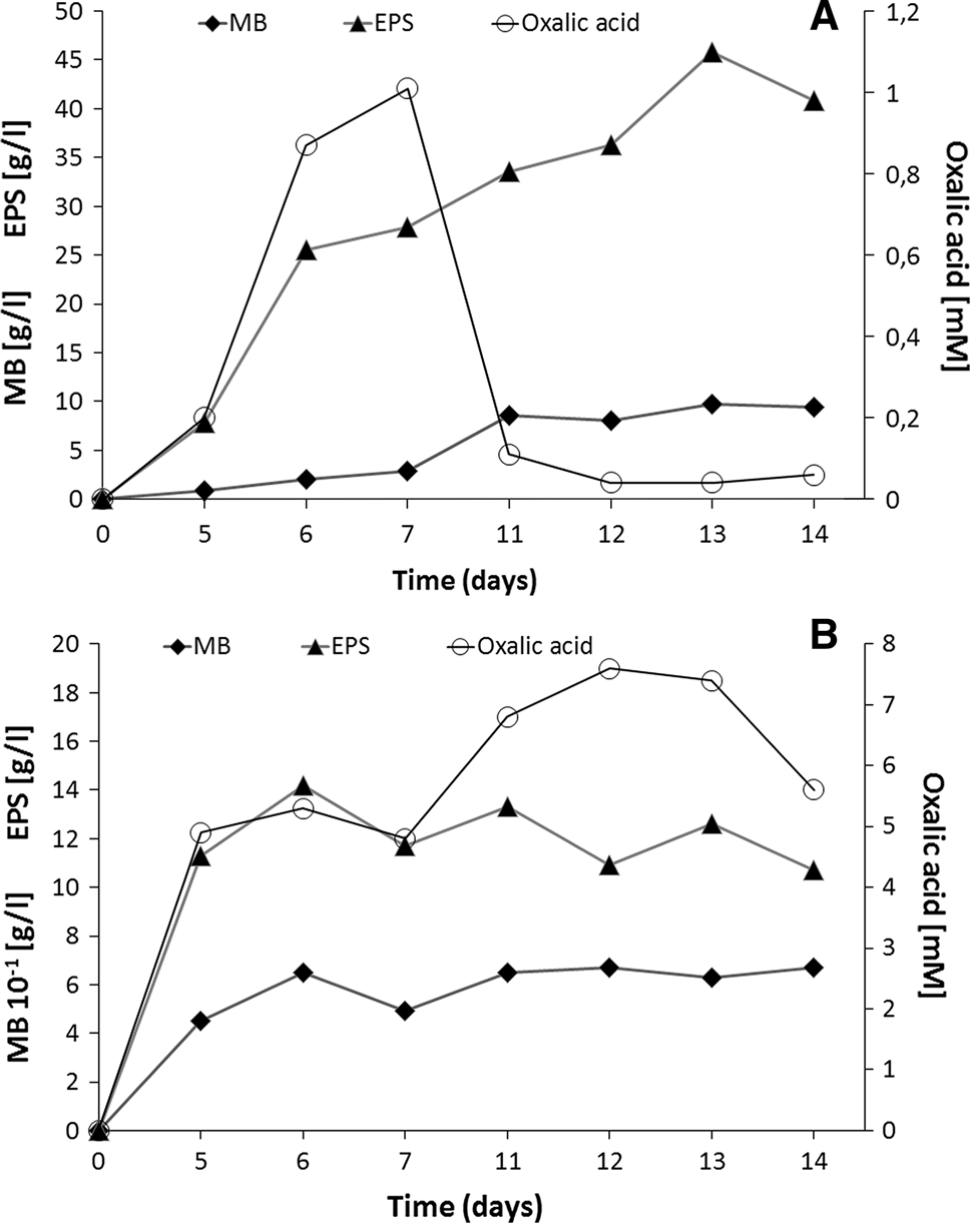
Changes in the dry weight of mycelial biomass (MB), the amount of exopolysaccharides (EPS), and the level of oxalic acid during the 14-day-long growth of *Ganoderma applanatum* (**a**) and *Tyromyces palustris* (**b**). Data are shown as mean ± SD (*p* < 0.01), (n = 3)

In order to determine the proper time for the production of exopolysaccharides (EPS), liquid cultures of *Ganoderma applanatum* (white rot strain) and *Tyromyces palustris* (brown rot strain) were performed during 14 days at 28 °C. During the period of fungal biomass cultivation, the level of exopolysaccharides and the level of oxalic acid were monitored and analysed (Fig. 1). Our results indicate that the production of exopolysaccharides during cultivation of *G. applanatum* increased with time and the highest concentration of exopolysaccharides was obtained on the 13th day of cultivation (45.8 ± 1.2 g/L). Also on that day, the culture achieved the maximum dry mass amounting to 9.72 ± 0.4 g/L (Fig. 1a). The concentration of extracellular oxalic acid reached the highest level (1.01 ± 0.2 mM) on the 7th day of *G. applanatum* growth. No direct correlation between the fungal growth, the amount of exopolysaccharide, and the concentration of oxalic acid in the culture medium was observed (Fig. 1a). The concentration of oxalic acid in *T. palustris*, the typical brown rot strain, was several-fold higher than that in *G. applanatum*, the typical white rot strain, and amounted to 7.6 ± 0.4 mM on the 12th day of growth (Fig. 1b). During the period of *T. palustris* growth, the high concentration of oxalic acid was correlated with the synthesis of exopolysaccharides (Fig. 1b).

#### Effect of cultivation temperature

**Fig. 2:**
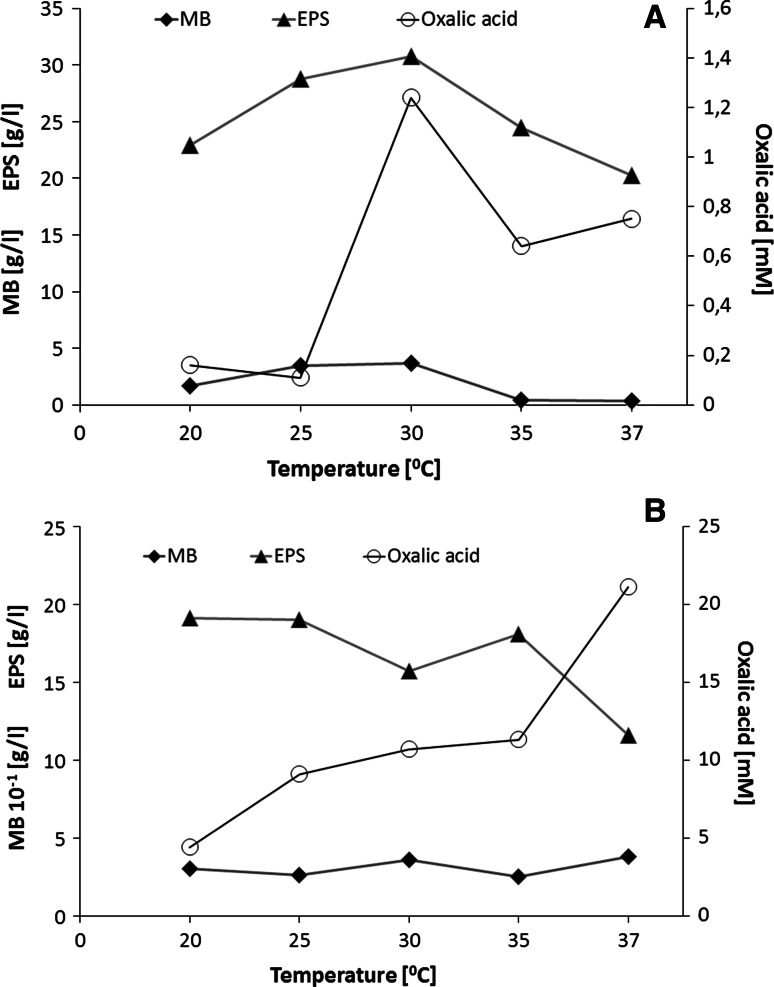
The effect of culture temperature on the amount of mycelial biomass (MB), the amount of exopolysaccharides (EPS), and the level of oxalic acid during the 10-day-long and 7-day-long growth of *Ganoderma applanatum* (**a**) and *Tyromyces palustris*, respectively (**b**). Data are shown as mean ± SD (*p* < 0.01), (n = 3)

**Fig. 3:**
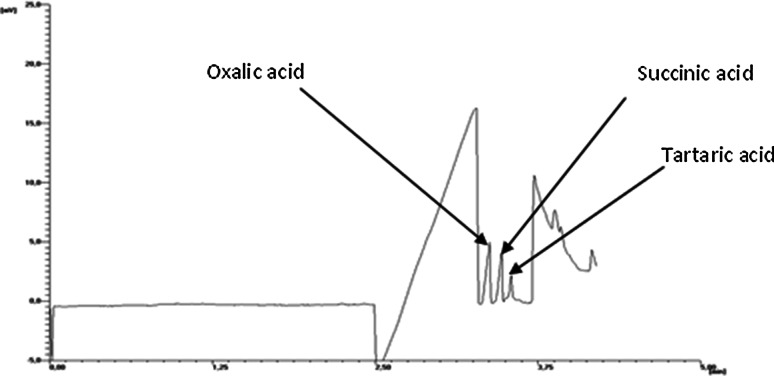
Capillary electrophoresis chromatogram of the growing medium obtained from *Ganoderma applanatum* cultures

To investigate the influence of temperature on the formation of fungal biomass and exopolysaccharides, the cultures of *G. applanatum* and *T. palustris* were carried out at various temperatures in the range from 20 to 37 °C for 10 and 7 days, respectively (the optimal culture time was experimentally confirmed in earlier investigations, data not shown). As shown in Fig. 2, both the maximum biomass formation and the exopolysaccharide production during cultivation of *G. applanatum* were observed during the growth at 28 °C, with the dry weight of biomass around 3.64 ± 0.2 g/L and exopolysaccharide yield reaching 30.78 ± 0.6 g/L. The optimal temperature for biomass formation during cultivation of *G. applanatum* corresponded with that for the production of exopolysaccharides and for the secretion of oxalic acid. At the same temperature, we also noticed the maximum level of oxalic acid (1.24 ± 0.2 mM) in the culture medium of *G. applanatum*. Apart from oxalic acid, the presence of succinic acid and tartaric acid was observed during the growth of *G. applanatum*, but these two acids were detected only in trace amounts (Fig. 3). The highest concentration of succinic acid (0.7 ± 0.01 mM) and tartaric acid (3.55 ± 0.03 mM) was observed only on the 11th day of cultivation of *G. applanatum* at 37 °C. *T. palustris* grew optimally in all the tested temperatures and the maximum dry weight of biomass was obtained at 37 °C (0.38 ± 0.02 g/L), while the maximum amount of exopolysaccharides was observed during the growth at a temperature of 20 °C (19.1 ± 1.2 g/L). It was observed that the concentration of oxalic acid in the culture media of *T. palustris* increased when the culture temperature increased from 20 to 37 °C, and the highest amount of oxalic acid was obtained in the cultures growing at 37 °C (21.1 ± 1.1 mM). The results indicate that the optimal temperature for the cell growth of *T. palustris* is not consistent with the optimal temperature for the secretion of oxalic acid.

#### Effect of the culture medium pH values

**Fig. 4:**
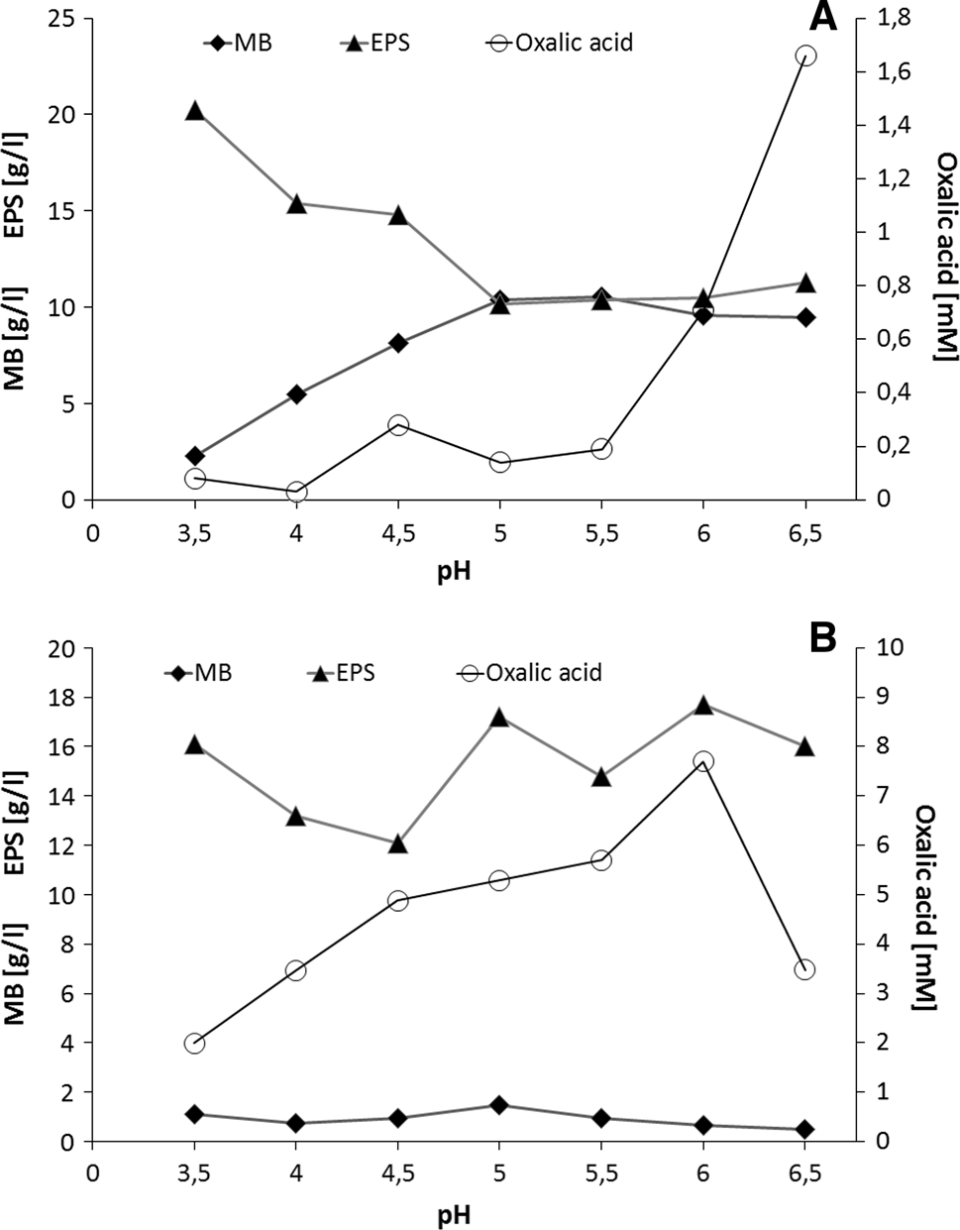
The effect of culture pH values on the amount of mycelial biomass (MB), the amount of exopolysaccharides (EPS), and the level of oxalic acid during the 7-day-long growth of *Ganoderma applanatum* (**a**) and *Tyromyces palustris* (**b**). Data are shown as mean ± SD (*p* < 0.01), (n = 3)

The influence of the initial pH values of the growing medium (in the range from 3.5 to 6.5) was tested during 7-day-long cultures carried out at a temperature of 28 °C (Fig. 4). An optimal initial pH value of the growing medium to obtain the maximum biomass of *G. applanatum* was pH 5.5 with biomass around 10.5 ± 0.5 g/L. The highest yield of extracellular polysaccharides was obtained during *G. applanatum* growth on a medium with pH 3.5, and the amount of exopolysaccharides was 20.23 ± 1.3 g/L. The secretion of oxalic acid by *G. applanatum* was correlated with the initial pH value of the growing medium and the highest concentration of oxalic acid was 1.66 ± 0.2 mM during the fungal growth at initial pH 6.5. In the case of *G. applanatum*, the correlation between the growing medium pH value and the secretion of both oxalic acid and exopolysaccharides was observed. The oxalic acid concentration increased at the higher pH values of the growing medium, while the yield of the exopolysaccharides increased at the lower initial pH values. This correlation was not observed during the growth of *T. palustris*, where the highest amounts of exopolysaccharides and fungal biomass production were obtained during the growth at the initial pH 5.0, while the reduction of the initial pH decreased the level of oxalic acid from 7.7 ± 0.6 mM at pH 6.0 to 1.99 ± 0.2 mM at pH 3.5 (Fig. 4b). The initial pH did not affect the final production of fungal biomass and exopolysaccharides during the growth of the brown rot *T. palustris* strain. During the fungal growth, acidification of the growing medium was observed in the case of both tested strains and the final pH of the cultures was from 3.3 to 3.6 for *G. applanatum* and from 2.6 to 2.7 for *T. palustris*.

#### Effect of the carbon source concentration

**Fig. 5:**
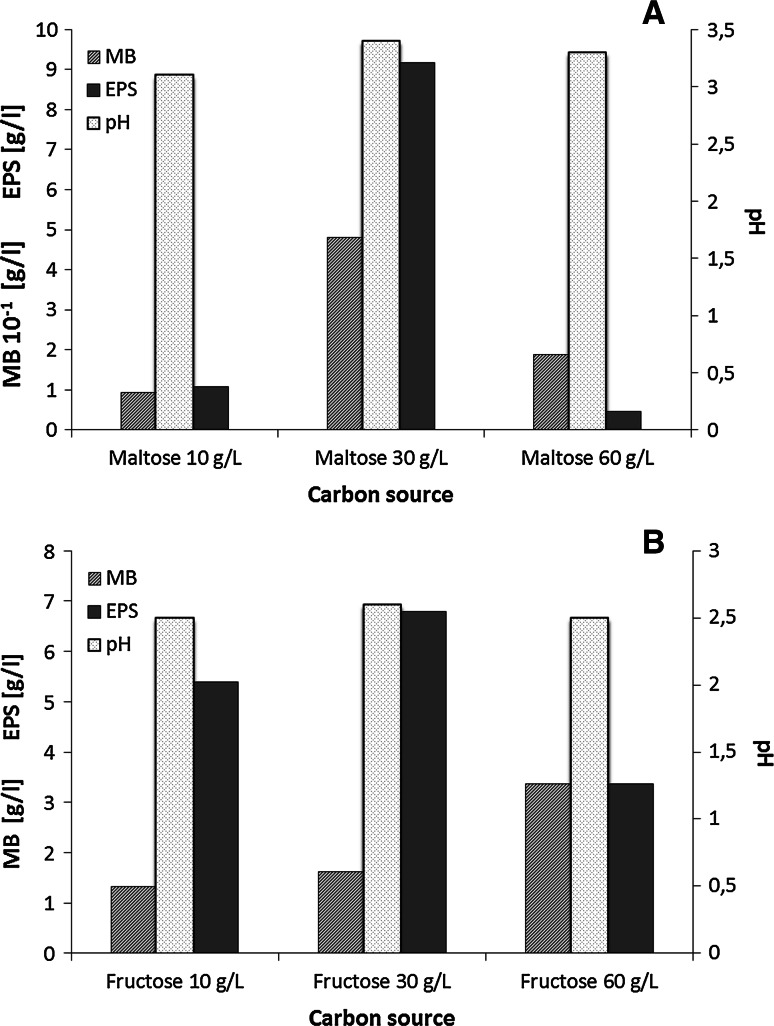
The effect of the carbon-source concentration on the amount of mycelial biomass (MB), the amount of exopolysaccharides (EPS), and the level of pH during the 7-day-long growth of *Ganoderma applanatum* (**a**) and *Tyromyces palustris* (**b**). Data are shown as mean ± SD (*p* < 0.01), (n = 3)

Five different carbon sources (glucose, maltose, sucrose, lactose, and fructose) were used to investigate their effect on the production of mycelial biomass and exopolysaccharide secretion. As shown in Table 1, the highest concentration of exopolysaccharides was obtained when maltose was used as a carbon source during cultivation of *G. applanatum.* It was observed during cultivation of *G. applanatum* that an increasing concentration of maltose used as a carbon source was correlated with the amount of fungal biomass (Fig. 5a). The best results for exopolysaccharides production (9.18 g/L) were obtained when the concentration of maltose was 30 g/L. In the case of *T. palustris,* the best carbon source was fructose. With an increasing concentration of this sugar, the mycelial biomass concentration increased to 3.36 g/L. A high yield of exopolysaccharide production (6.8 g/L) was observed during fungal growth in a medium containing 30 g/L fructose as the main carbon source (Fig. 5b).

#### Properties of crude exopolysaccharides

Crude exopolysaccharides of *T. palustris* and *G. applanatum* were extracted by addition of 95 % ethanol to fermentation broth filtrates. The contents of total polysaccharides and reducing sugars in the preparation of *T. palustris* were analysed and compared with values described for *G. applanatum* exopolysaccharides (Osińska-Jaroszuk et al. 2014). The total carbohydrate content of the exopolysaccharide extracts varied considerably from 303 mg/g dwc (dry weight of crude polysaccharide) of the extract (30.3 %) for *G. applanatum* to 54.1 mg/g dwc (5.4 %) for *T. palustris* (Table 2). The low yield of EPS from the *T. palustris* culture may be explained by the weak growth of this strain and, consequently, a low amount of biomass. The exopolysaccharide extracted from *T. palustris* showed the presence of reducing sugars (4.03 ± 0.2 mg/g dwc), uronic acids (0.13 ± 0.01 mg/g dwc), and proteins (0.15 ± 0.01 mg/g dwc), and these values are compared in Table 2 to the values for the exopolysaccharide extracted from *G. applanatum* (Osińska-Jaroszuk et al. 2014). These results indicate that the protein contents of crude exopolysaccharide extracts both from *G. applanatum* and from *T. palustris* were very low and amounted to 10 % and 0.3 % respectively. Hexosamines were detected in both fungal exopolysaccharides and it was 0.31 ± 0.01 mg/g dwc in the case of the exopolysaccharide from *G. applanatum* and 0.04 ± 0.02 mg/g dwc in the case of the exopolysaccharide from *T. palustris* (Table 2).

**Table 1:** Amount of crude exopolysaccharides (EPS) produced by *G. applanatum* and *T. palustris* during the growth on media with different sugars used as the main carbon source (30 g/L)

Carbon source (30 g/L)	EPS produced by *G. applanatum* ^a^ (mg/g)	EPS produced by *T. palustris* ^a^ (mg/g)
Glucose	312	121.3
Sucrose	100.1	698.4
Fructose	119.9	1,597.7
Lactose	134.8	138.9
Maltose	320	0

^a^Weight of crude exopolysaccharide produced by 1 g of mycelium

**Table 2:** Amount of protein, carbohydrate, total polysaccharides, reducing sugars, uronic acids, and hexosamines in crude polysaccharide of *Tyromyces palustris* (n = 3) in comparison to exopolysacharide of *Ganoderma applanatum* (Osińska-Jaroszuk et al. 2014)

Crude polysaccharide	Protein content^a^ (mg/g)	Total carbohydrate^a^ (mg/g)	Total polysaccharide^a^ (mg/g)	Reducing sugar^a^ (mg/g)	Uronic acid (mg/g)	Hexosamines (mg/g)
*Tyromyces palustris*	0.15 ± 0.01	54.1 ± 1.3	50.13 ± 1.0	4.03 ± 0.2	0.13 ± 0.01	0.04 ± 0.02
*Ganoderma applanatum*	22.6 ± 0.07^b^	303.0 ± 1.29^b^	214.8 ± 2.0^b^	61.2 ± 1.2^b^	2.1 ± 0.1	0.31 ± 0.01

^a^Per gram of crude polysaccharide dry weight

^b^Osińska-Jaroszuk et al. (2014)

**Fig. 6:**
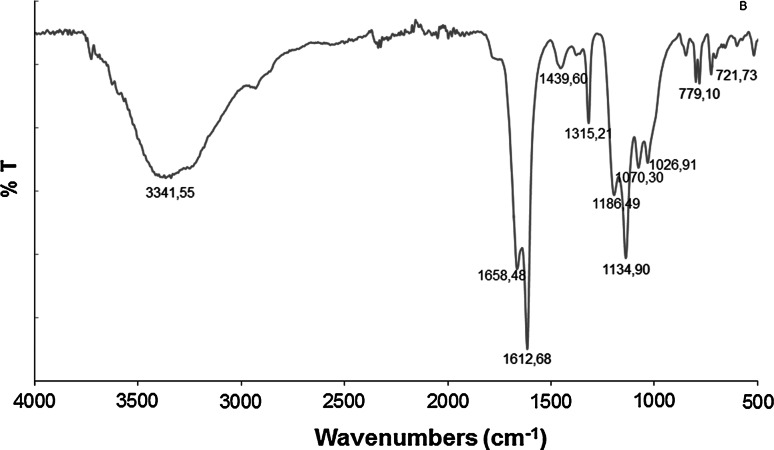
FT-IR spectra of an exopolysaccharide obtained from the *Tyromyces palustris* cultures

As shown in Fig. 6, the FT-IR spectrum of the exopolysaccharide from the brown rot *T. palustris* strain exhibited a band at 3,342.5 cm^−1^ characteristic for the hydroxyl group. A small amount of protein was also observed with absorptions at 1,612.6 and 1,439 cm^−1^ (Carey 1992; Šandula et al. 1999). Other bands related to the presence of β-glucans were found near 1,134, 1,070.3, and 1,026.9 cm^−1^ (Gutièrrez et al. 1996; Šandula et al. 1999). Additionally, the spectrum of exopolysaccharides from *T. palustris* showed a band at 1,315.2 cm^−1^ suggesting presence of the –CH = group in the sugar ring. The FT-IR spectrum of ethanol-extracted exopolysaccharides of the other strain, *G. applanatum,* was published in our earlier paper (Osińska-Jaroszuk et al. 2014). The FT-IR spectrum analysis showed presence of the hydroxyl group (OH), carboxylic group (COOH), and C–O bands characteristic for presence of β-glucans.

## Discussion

Due to their functions, polysaccharides are widespread in the world of fungi. As biologically active particles, they are the subject of many studies designed to provide exact knowledge of their action and influence on living organisms (Russel and Paterson 2006). It is known that the ability to produce exopolysaccharides by fungal cultures is related to the type of substrates on which these cultures are growing. Both the composition of the culture medium and environmental conditions have an influence on the properties of polysaccharides obtained. It is believed that depending on the culture variants used derived polysaccharide preparations can differ in the degree of polymerization, mechanical strength, and possible application.

Our studies have shown that both glucose and maltose used as carbon sources are effectively used by *Ganoderma apllanatum* for growth and production of exopolysaccharides. The highest concentration of the exopolysaccharide was observed during fungal growth on the medium with maltose at a concentration of 30 g/L. In the case of higher concentrations of maltose, an increase of biomass weight and reduction of exopolysaccharide secretion was observed. The lower concentration of maltose affected the growth of the mycelium and polysaccharide production. Lee et al. (2007) showed that a high yield of exopolysaccharide production by *G. applanatum* required a high glucose concentration (60 g/L). Similarly, during *Ganoderma lucidium* cultivation, the concentration of EPS (1.79 g/L) was optimal when the concentration of glucose increased to 60 g/L (Hsieh et al. 2006). Smiderle et al. (2012) observed a number of alternative carbon sources for production of exopolysaccharides by a *Pleurotus pulmonarius* strain. Irrespective of the carbon source used, similar levels of polysaccharide production were achieved and the type of the sugar used had a significant influence on the composition of the polymer obtained. It was observed that *Tyromyces palustris* growing on glucose medium produced large amounts of oxalic acid which affected the production of polysaccharides (Xiong et al. 2007). Therefore, as we have shown in our research, fructose was a better carbon source for obtaining larger quantities of exopolysaccharides from the *T. palustris* strain.

Oxalic acid was the main organic acid secreted by the tested strains, as has been reported earlier for other wood-rotting strains, both white rot (Jarosz-Wilkołazka and Grąz 2006) and brown rot (Shimada et al. 1997). Cultures of brown rot fungi often contain substantial amounts of oxalic acid, whereas cultures of white rot fungi usually have little or no oxalic acid (Shimada et al. 1997). Akamatsu et al. (1994) screened 24 strains (both brown and white rot fungi) for oxalic acid production on low and high nitrogen media. Among the brown rot fungi, the highest concentration of oxalic acid was found in low nitrogen media for *Tyromyces palustris* (47.5 mM), *Coniophora puteana* (15.1 mM), and *Postia placenta* (18.3 mM). White rot fungal species, for example *Coriolus versicolor* (4.0 mM), *Pleurotus ostreatus* (27.8 mM), *Phanerochaete chrysosporium* (5.8 mM), and *Ganoderma applanatum* (1.4 mM) preferred higher concentrations of nitrogen sources in cultures to obtain higher concentrations of oxalic acid. Additionally, Kaneko et al. (2005) showed that brown rot fungi such as *Tyromyces palustris*, *Laetiporus sulphurous*, and *Postia placenta* accumulated considerable amounts of oxalic acid during their growth on media with a different composition and the maximum concentration of oxalic acid was reached after 20 days of fungal growth. The authors suggest that oxalic acid may be involved in hydroxyl radical (^·^OH) production by typical brown rot fungi that have substantial wood-degrading activities (Kaneko et al. 2005).

In our earlier paper, oxalic acid was identified as the main and important metabolite produced in response of white rot fungi to the presence of metallic oxides (Jarosz-Wilkolazka and Graz 2006). We reported that *G. applanatum* was highly tolerant to the tested metal oxides and this tolerance was correlated with the secretion of oxalic acid. Additionally, during the growth of *G. applanatum* in the presence of the tested metal oxides, significantly lower (acidic) pH of the growing medium was observed, which was correlated with the secretion of oxalic acid (Jarosz-Wilkolazka and Graz 2006). In this study, we observed that with the increasing baseline pH of the medium, during the culture time of *T. palustris* and *G. applanatum* the pH value decreased to a value of approx. 2.5 and 3.5, respectively. This observation is consistent with scleroglucan production by *Sclerotium rolfsii* described by Schilling et al. (2000), who reported that at initial pH 2.0 only traces of oxalic acid were detected in the culture medium, while scleroglucan production remained unchanged. On the other hand, Shu and Lung (2004) showed that the optimal pH for biomass formation during cultivation of *Antroida camphorata* was around 4.0 and for exopolysacharide production around 5.0. The different optimal pH values for the same culture parameters tested in different fungal strains may be due to the differences in the ecological type of strains, the composition of fermentation media, and the culture conditions. Lee et al. (2007) showed that a short period of culturing (8–12 days) and moderate temperatures (25 °C) favoured the production of exopolysaccharides by *G. applanatum.* The most efficient production of pullulan synthesised from *Aureobasidium pullulans* was observed in the pH range between 5.0 and 7.5 and a temperature of 20–26 °C (Wu et al. 2012). Production of scleroglucan by *Sclerotium glucanicum* is most intense at 28 °C. Above this temperature, high oxalic acid synthesis was noted (Wang and McNeil 1995).

The *Tyromyces palustris* strain is known for production of large amounts of oxalic acid, which is probably due to the lack of synthesis of enzymes that can break down this acid (Watanabe et al. 2010). Our results showed that the *G. applanatum* strain (white rot fungus) accumulated ca. 20-fold less oxalic acid in comparison with *T. palustris* (brown rot fungus), and this observation was in agreement with the results reported by Shimada et al. (1997). However, literature provides data that fungal strains belonging to white rot degraders e.g. *Abortiporus biennis* and *Cerrena unicolor* produce high concentrations of oxalic acid (Jarosz-Wilkołazka and Grąz 2006). Presence of oxalic acid was observed in the liquid culture media in both fungal strains studied (*G. applanatum* and *T. palustris*). It was found that the amount of oxalic acid produced is correlated with the initial pH value of the culture medium. Along with an increase in pH values, the synthesis and secretion of oxalic acid into the intercellular space was increased. It was also found that the increased secretion of oxalic acid depended on the cultivation temperature. In their studies on the synthesis of scleroglucan by *Sclerotium glucanicum*, Wang and McNeil (1995) confirmed that the amount of secreted oxalic acid increased linearly with the increased medium pH above the value of 3.5. Probably, this is associated with stimulation of the enzyme synthesizing oxalic acid. An important problem in exopolysaccharide production is the fact that the formation of oxalic acid in fungal cells is associated with the synthesis of the carbon sources that could be used for the production of exopolysaccharides (Survase et al. 2007). Increased synthesis of polysaccharides can be obtained by controlling the pH during the growth and synthesis phases, which leads to strong inhibition of oxalate. Galkin et al. (1998) studied fifteen white rot fungi cultured in liquid and solid ligninocellulose media and found, in addition to oxalic acid, also malic, malonic, and tartaric acids. In our earlier paper, we showed that oxalic acid was the main organic acid secreted by the tested white rot strains to a solid medium amended with metal oxalates (Jarosz-Wilkolazka and Graz 2006), but formic acid and malic acid were also detected in cultures of some strains. Only two strains, *Pholiota nameko* and *Pleurotus cystidiosus,* of all the tested white rot strains did not produce oxalic acid during growth on ZnO-amended plates (Jarosz-Wilkolazka and Graz 2006).

The amounts of total polysaccharides of the *G. applanatum* cultures were higher than the amounts reported in crude, hot-water-extracted polysaccharides from *Ganoderma applanatum* and *Ganoderma lucidium*, which were 20 and 30 % of polysaccharides, respectively (Kozarski et al. 2011; Osińska-Jaroszuk et al. 2014). Telles et al. (2011) found 22.6 % of total carbohydrates in native extracellular polysaccharides from *Pleurotus sajor*-*caju*. However, a high content of total sugar was shown in exopolysaccharides from *Cordyceps sinensis* comprising from 46 to 70 % of polysaccharides and it depended on the day of the growth (Leung et al. 2009). The polysaccharide fractions from another strain of the *Ganoderma* genus*, G. lucidium*, also contained protein (ca. 6.71 %) and were characterized as glycopeptides (Jia et al. 2009) The crude exopolysaccharide isolated from the mycelium of *Cordyceps sinensis* described by Leung et al. (2009) contained 65–75 % of sugar and ca. 25 % of protein suggesting a polysaccharide-protein character. Cui and Chisti (2003) reported that a polysaccharide-peptide complex from *Coriolus versicolor* contained peptide mainly consisting of aspartic and glutamic acids. Our results showed that exopolysaccharides obtained both from *G. applanatum* and *T. palustris* additionally possessed hexosamines and uronic acids.

In conclusion, it is worth noting that the initial pH values of the growing medium for *G. applanatum* and *T. palustris* cultivation have an influence on mycelium growth, exopolysaccharide formation, and oxalic acid production. The optimal pH value for exopolysaccharide formation was lower for *G. applanatum* (white rot strain) than for *T. palustris* (brown rot strain), which can suggest natural pH reduction by oxalic acid secretion in the case of the brown rot strain. During the cultivation of the white rot *G. applanatum* strain, the concentration of secreted oxalic acid increased during the growth on a medium with a higher pH value. This correlation was not observed during the cultivation of *T. palustris*.

In addition, we observed that the optimal temperature for *G. applanatum* growth was correlated with exopolysaccharide and oxalic acid production and no such correlation was observed in the case of cell growth and oxalic acid secretion in the case of *T. palustris*. The results obtained from this study could be used to understand the correlation between exopolysaccharide formation and oxalic acid production during the growth of fungal strains belonging to ecologically different groups, i.e. white rot (*Ganoderma applanatum*) and brown rot (*Tyromyces palustris*) degraders. This is very important especially for fungal strains which produce polysaccharides with biomedical properties such as *Ganoderma applanatum*. Recently, an exopolysaccharide isolated from *G. applanatum* has been shown to have many biomedical, e.g. anti-cancer, immunomodulating, and antibacterial properties (Osińska-Jaroszuk et al. 2014). The knowledge about the production and secretion of fungal exopolysaccharides should be extended to find out fungal strains with extraordinary physiological and biotechnological properties.
